# Stay Mindful and Carry on: Mindfulness Neutralizes COVID-19 Stressors on Work Engagement *via* Sleep Duration

**DOI:** 10.3389/fpsyg.2020.610156

**Published:** 2020-12-21

**Authors:** Michelle Xue Zheng, Theodore Charles Masters-Waage, Jingxian Yao, Yizhen Lu, Noriko Tan, Jayanth Narayanan

**Affiliations:** ^1^Department of Organizational Behavior and Human Resource Management, China Europe International Business School (CEIBS), Shanghai, China; ^2^NUS Business School, Singapore Management University, Singapore, Singapore; ^3^Católica Lisbon School of Business and Economics, Catholic University of Portugal, Lisbon, Portugal; ^4^NUS Business School, National University of Singapore, Singapore, Singapore

**Keywords:** mindfulness, COVID-19 stressors, employee sleep, work engagement, organizational behavior

## Abstract

We examine whether mindfulness can neutralize the negative impact of COVID-19 stressors on employees’ sleep duration and work engagement. In Study 1, we conducted a field experiment in Wuhan, China during the lockdown between February 20, 2020, and March 2, 2020, in which we induced state mindfulness by randomly assigning participants to either a daily mindfulness practice or a daily mind-wandering practice. Results showed that the sleep duration of participants in the mindfulness condition, compared with the control condition, was less impacted by COVID-19 stressors (i.e., the increase of infections in the community). In Study 2, in a 10-day daily diary study in the United Kingdom between June 8, 2020, and June 19, 2020, we replicate our results from Study 1 using a subjective measure of COVID-19 stressors and a daily measure of state mindfulness. In addition, we find that mindfulness buffers the negative effect of COVID-19 stressors on work engagement mediated by sleep duration. As the COVID-19 pandemic is ongoing and the number of reported cases continues to rise globally, our findings suggest that mindfulness is an evidence-based practice that can effectively neutralize the negative effect of COVID-19 stressors on sleep and work outcomes. The findings of the present study contribute to the employee stress and well-being literature as well as the emerging organizational research on mindfulness.

## Introduction

Sleep helps employees recover from work and restore their resources (Hülsheger et al., 2014, 2015; Steed et al., 2019). Short sleep duration is associated with detrimental physical health outcomes such as cardiovascular diseases, coronary heart diseases, and even mortality (see Itani et al., 2017, for a review). Low levels of sleep also have destructive effects on psychological outcomes such as cognitive performance (Lim and Dinges, 2010), neurocognitive functioning (Durmer and Dinges, 2005), and mental health (Benca et al., 1992). Importantly, employees with insufficient sleep feel depleted in the workplace, are less satisfied with their jobs, exhibit less organizational citizenship behaviors, and have poor work performance (Kessler et al., 2011; Barnes et al., 2012, 2013; Lanaj et al., 2014; see Litwiller et al., 2017, for a review).

A prominent downstream effect of short sleep duration in the workplace is impaired work engagement (Lanaj et al., 2014; Litwiller et al., 2017). Work engagement is a powerful predictor of employees’ job performance. Extant research has shown that more engaged employees perform better in the workplace (Rich et al., 2010; Christian et al., 2011; Bakker et al., 2012; Van Wingerden et al., 2017). Employees who are highly engaged in their work activities not only devote their physical effort, but are also mentally vigilant and emotionally dedicated to the endeavor, and thereby performing better in their tasks (Kahn, 1990; Ashforth and Humphrey, 1995; Rich et al., 2010).

Given that poor sleep takes a serious toll on employees and organizations, organizational research has identified organizational antecedents that impede employees’ sleep (Litwiller et al., 2017). Previous studies have shown that demands such as occupational stressors (DeArmond and Chen, 2004), employees’ late night smartphone use for work (Lanaj et al., 2014), long hours worked per week (Blau, 2011), and work-family conflict (Barnes et al., 2012; Berkman et al., 2015) are antecedents of employees’ reduced sleep duration. Although it is important to understand organizational factors that keep employees awake at night, sleep as a recovery process may also be influenced by non-work factors. In fact, it has long been recognized that organizational scholars should also consider the potential role of non-organizational factors in studying employees’ recovery process (Sonnentag, 2003; see Steed et al., 2019 for a recent review).

Exposure to traumatic events is probably the most powerful non-organizational factor that disturbs employees’ sleep (Lavie, 2001; see Sinha, 2016, for a review). The ongoing COVID-19 pandemic is the defining global crisis of our time. The UN has referred to it as the greatest challenge humanity has faced since the II World War (United Nations, 2020). Even as we prepare this manuscript, the situation is evolving with cases rising daily in Africa, the United States, and Europe. Scholars have drawn attention to the impacts it could have on individuals’ psychological well-being and functioning, with some scholars referring to it as a “collective trauma” (Silver, 2020; Van Bavel et al., 2020; Kniffin et al., In press). Organizational scholars have termed this type of large-scale traumatic events as *acute-extraorganizational stressors* (Byron and Peterson, 2002; Hochwarter et al., 2008). The defining feature of an acute-extraorganizational stressor is that it is driven by a sudden or extreme force that is external to organizations. Unlike intra-organizational stressors (e.g., organizational restructuring or high work demands), organizations cannot take active steps to prevent stressors induced by COVID-19. Scholars have argued that such extra-organizational stressors can have a more devastating effect on employees than intra-organizational stressors (Byron and Peterson, 2002; Hochwarter et al., 2008). Nevertheless, organizations can take intervening steps to mitigate the negative effects of acute-extraorganizational stressors on their employees. As such, it is important for organizational scholars to examine factors that can protect employees from these negative effects during the crisis. However, to date, research has mainly focused on intra-organizational stressors, and thus, we have limited understanding about extra-organizational stressors (Hochwarter et al., 2008). Given this dearth of research, scholars have called for studies that provide theoretical and practical recommendations for how organizations can help their employees manage acute-extraorganizational stressors (Byron and Peterson, 2002; Hochwarter et al., 2008; James, 2011).

We build on the metacognitive theory of mindfulness (Ong et al., 2012; Jankowski and Holas, 2014) and the recovery literature (Barnes, 2012; Steed et al., 2019) to propose that mindfulness can neutralize the negative effect of COVID-19 stressors on employee sleep and employee work engagement (Schaufeli et al., 2008). Specifically, given that exposure to trauma can stimulate an intense and sustained state of hyperarousal, which, in turn, disrupts individuals’ sleep (Lavie, 2001; Germain, 2013; Sinha, 2016), we argue that mindfulness – a state in which individuals become aware of their present moment experience – can reduce this hyperarousal state and thereby mitigate the negative effect on sleep duration. Further, given that sleep duration is a crucial recovery mechanism that leads to more engaged employees at work (Barnes, 2012; Lanaj et al., 2014), we propose that state mindfulness may be able to neutralize the negative effects of the COVID-19 stressors on work engagement through the mediating role of sleep duration.

## Covid-19 Stressors, Sleep Duration, and Work Engagement

Sleep disruption is a prominent feature of individuals’ neurobiological and physiological response to trauma (Sinha, 2016). Trauma generates a stressful response that leads to physiological hyperarousal, which in turn disrupts sleep (Lavie, 2001; Germain, 2013; Sinha, 2016). The hyperarousal state occurs at two levels: primary arousal and secondary arousal (Ong et al., 2012). Primary arousal refers to cognitive activities that directly impair sleep, such as worrying about the impact of COVID-19. Secondary or metacognitive arousal refers to the awareness and judgment of primary arousal (i.e., thinking about thinking), which includes how negatively individuals evaluate their thoughts that occurred at the primary level. For example, people may further ruminate about their stressful thoughts about COVID-19 and amplify a hyperarousal state. They may become more attentive to and obsessed with the thoughts that occur at the primary level, which may result in a vicious cycle of falling and/or staying asleep. Indeed, research has shown that exposure to traumatic events leads to shorter sleep duration (Sinha, 2016; Goodwin et al., 2018). Thus, it is possible that employees will experience sleep disruption in response to COVID-19 stressors.

Returning to work after a good night’s sleep is critical to ensure employees have sufficient energy and self-regulatory resources to work (Barnes, 2012) and helps employees achieve psychological detachment and physiological recovery (Steed et al., 2019). Thus, sleep is a crucial recovery mechanism leading to work engagement (Barnes, 2012; Lanaj et al., 2014). Specifically, work engagement is defined as a cognitive-affective state characterized as being vigorous, dedicated, and absorbed in work (Schaufeli et al., 2002; Schaufeli and Bakker, 2004). Engaged employees have high energy, are intensely involved, and are enthusiastic and immersed in work activities. In line with our theorizing, past research has established that sleep duration is positively associated with work engagement (Lanaj et al., 2014; Litwiller et al., 2017).

Given that employees react to trauma by losing sleep (Lavie, 2001; Germain, 2013; Sinha, 2016) and that sleep duration is a crucial recovery mechanism leading to work engagement (Barnes, 2012; Lanaj et al., 2014; Litwiller et al., 2017), we argue that COVID-19 stressors may damage employees’ work engagement *via* impaired sleep duration. However, previous research has suggested that these stressors do not universally impact employees (Hochwarter et al., 2008). For example, Hochwarter et al. (2008) have found that employees’ perceived resources interact with the effect of hurricane induced stressors on job satisfaction such that hurricane stress reduces job satisfaction for employees with lower perceived resources while hurricane stress is neutralized for employees with higher perceived resources. Thus, there are boundary conditions that determine whether acute-extraorganizational stressors negatively impact employees. Building on the metacognitive theory of mindfulness which suggests that mindfulness is effective in reducing hyperarousal state and improves sleep (Ong et al., 2012; Jankowski and Holas, 2014), we propose that mindfulness is a boundary condition that buffers the negative effect of COVID-19 stressors on sleep duration and in turn work engagement.

## Mindfulness Neutralizes the Negative Effect of Covid-19 Stressors

Mindfulness is defined as a moment-to-moment non-judgmental awareness of one’s present experience (Brown and Ryan, 2003). Mindfulness can be viewed as a naturally occurring mental state (measured as a dispositional trait or a transient mental state) or can be trained through meditation practices (Davidson, 2010). Despite these distinct operationalizations, scholars view the mindfulness state as a unitary construct across these measures (Reb and Atkins, 2015; Good et al., 2016). This state of mind has been linked with numerous positive outcomes, such as reduced employee stress (for a meta-analysis see Bartlett et al., 2019), and outcomes more specific to the workplace (for reviews see Reb and Atkins, 2015; Good et al., 2016; Eby et al., 2019). Existing research suggests that many of these benefits are a result of mindfulness, increasing a metacognitive awareness of one’s experience (Jankowski and Holas, 2014; Kay and Skarlicki, 2020). Specifically, the metacognitive theory of mindfulness suggests that a non-judgmental awareness of one’s present experience facilitates individuals’ capacity to observe their experience as something separate from themselves. By generating psychological distance between oneself and one’s immediate experience, mindfulness supports individuals’ capacity to observe and to accept their thoughts and experiences without judgments (Jankowski and Holas, 2014).

As discussed above, sleep is disrupted because metacognitive arousal amplifies the primary arousal triggered by COVID-19 stressors. Mindfulness can specifically mitigate the metacognitive arousal by shifting the negative metacognitive process to a more adaptive stance, in which individuals simply observe and accept their primary thoughts without judgments (Ong et al., 2012; Jankowski and Holas, 2014). In other words, mindfulness may prevent a primary arousal state from developing into a secondary (metacognitive) arousal state. As a result, mindful employees have less difficulty falling asleep and, thus, experience a longer sleep duration. Indeed, previous studies have established that mindfulness effectively increases sleep quantity (Hülsheger et al., 2015; see Ong and Smith, 2017, for a review). Therefore, building on the metacognitive theory of mindfulness and previous studies, we argue that mindfulness may be effective in buffering the negative effects of COVID-19 stressors on sleep duration. Thus, we propose the following hypothesis,

***Hypothesis*** 1: COVID-19 stressors interacts with mindfulness to predict sleep duration such that COVID-19 stressors negatively affects sleep duration when mindfulness is low while the effect of COVID-19 stressors is buffered when mindfulness is high.

**Figure 1:**
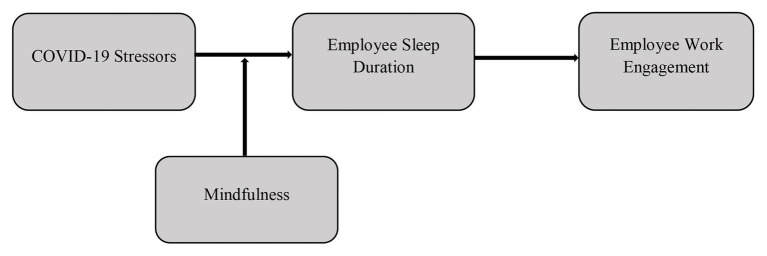
Conceptual model.

Building on the preceding hypothesis that argues that mindfulness will neutralize the negative effect of COVID-19 stressors on sleep duration and previous evidence that sleep duration is a key recovery resource leading to work engagement (Lanaj et al., 2014; Litwiller et al., 2017), we further propose that the buffering effect of mindfulness on the relationship between COVID-19 stressors and work engagement is mediated by sleep duration (see Figure 1).

***Hypothesis*** 2: The interaction effect of COVID-19 stressors and mindfulness on work engagement is mediated by sleep duration.

## Overview of Studies

We tested our hypotheses in two studies. We tested hypothesis 1 in Study 1 which is a 10-day field experiment conducted among employees in Wuhan, China during the lockdown between February 20, 2020, and March 2, 2020. We operationalized COVID-19 stressors as an objective indicator ‐ the increase of infections in the community as this was salient to inhabitants in Wuhan at that time. Participants in this study were randomly assigned to either a daily mindfulness practice or a daily mind-wandering practice. Study 2 is a 10-day longitudinal survey conducted among employees in the United Kingdom between June 8, 2020 and June 19, 2020. This study serves two purposes. First, we increased the ecological validity of our research by replicating the buffering effect of mindfulness in a different country where the COVID-19 pandemic had spread widely within the country. Second, we provided additional robustness to our model by adopting alternative operationalizations of our primary independent variables, testing the moderated mediation model by measuring self-reported state mindfulness as a within-subject individual difference variable and COVID-19 stressors as employees’ self-reported variable.

## Study 1

### Participants

A snowballing technique was employed to recruit participants through an online advertisement posted through the first author’s personal networks in Wuhan between February 18, 2020 and February 20, 2020. The ad stated that a study was recruiting working adults who were experiencing the lockdown in Wuhan to complete a 12-day study with a compensation of 100 RMB (approximately USD 14). All procedures in the study were in accordance with the ethical standards of the institutional review board at the first author’s institution and with the Declaration of Helsinki, 1964, and its later amendments or comparable ethical standards. One day before the daily surveys, participants completed a consent form online. Subject IDs were assigned upon submitting the consent form in which a computerized random number was generated at the end of the form. To ensure anonymity, we did not ask participants to indicate their names throughout the study. All participants were blind to the study condition. Throughout the study, the research assistant used the subject ID to identify participants.

To ensure randomization, the research assistant who managed the study was blind to the treatment and the hypothesis. Interested participants scanned a QR on WeChat to enroll in the study. Participants were randomly assigned to one of the two WeChat anonymous groups. Recruitment stopped when the goal of enrolling 110 participants was reached. Experimental conditions were randomly assigned to these two groups by the first author. There are 60 participants in the mindfulness condition and 50 participants in the mind-wandering condition. Three participants in the mindfulness condition and six participants in the mind-wandering condition did not complete the daily surveys. Thus, they were not included in the final analyses. The response rate of initially enrolled participants to daily surveys did not significantly differ between the mindfulness and the mind-wandering groups [95% mindfulness, 88% mind-wandering, *χ*^2^ (1) = 1.78, *p* = 0.18]. The survey App automatically timestamped the initiation and the completion of the morning survey. This timestamp was used to check if participants adhered to their daily practice. Among participants who completed the 12-day study, one participant in the mindfulness condition and two participants in the mind-wandering condition did not practice the exercise in the morning for at least four consecutive days. In addition, one participant in the mindfulness condition was not located in Wuhan; since our study coded for information about new cases in Wuhan, we dropped this participant from the analysis as well. After excluding participants who did not meet the study criteria, we obtained a final sample of 97 with 55 participants remaining in the mindfulness condition and 42 in the mind-wandering condition. The sample size in the two conditions is comparable with previous mindfulness research (Lindsay et al., 2019; Hafenbrack et al., 2020). The 97 participants (68.04% female) have an average age of 34.49 years (*SD* = 5.03), 79.4% have a college/Bachelor’s degree, and 21.6% have a Masters/PhD degree. Participants in the mindfulness and the mind-wandering conditions did not significantly differ in terms of their sociodemographic features. Specifically, the distribution of gender in the mindfulness condition (58.2% female) did not differ from those in the mind-wandering condition (76.2% female), *χ*^2^ (1) = 2.58, *p* = 0.064. Participants’ age in the mindfulness condition (34.69 years, *SD* = 5.66) did not differ from those in the mind-wandering condition (34.33 years, *SD* = 4.03), *F*(1, 96) = 0.39, *p* = 0.54. There was no difference between mindfulness condition (27.69 years, *SD* = 12.42) and mind-wandering condition (28.36 years, *SD* = 10.85) in terms of years living in Wuhan, *F*(1, 96) = 0.13, *p* = 0.73. Participants in both conditions share similar education level (bachelor degree or above 80% vs. 90.5%), *χ*^2^ (1) = 0.71, *p* = 0.40. In addition, they did not differ in terms of trait mindfulness (4.82, *SD* = 0.54 vs. 4.83, *SD* = 0.83), *F*(1, 96) = 0.05, *p* = 0.83. These participants in the final sample also did not differ from individuals who initially enrolled in the study but failed to actually participate in the daily surveys (gender: *χ*^2^ (1) = 0.002, *p* = 0.97.; age: *F*(1, 105) = 2.36, *p* = 0.13, years living in Wuhan (*F*(1, 105) = 0.34, *p* = 0.56, trait mindfulness *F*(1, 105) = 0.53, *p* = 0.47). Although there was a marginally lower percentage of women in the mindfulness condition, this was the result of the snowballing technique rather than the planned assignment. As a robustness check, we controlled for gender in our analyses and found that the buffering effect of mindfulness on the relationship between daily confirmed cases and sleep quantity remained significant (*B* = 0.06, *SE* = 0.03, *p* = 0.02). We found no effect of gender (*B* = 0.01 *SE* = 0.03, *p* = 0.74).

### Procedure

Participants completed a baseline assessment on February 20, 2020, that asked for their demographic information and trait mindfulness a day before the intervention began. Participants in the mindfulness practice condition engaged in a 10-min mindfulness practice each morning and participants in the mind-wandering condition engaged in a 10-min mind-wandering practice for 10 consecutive days from February 21, 2020, (Friday) to March 1, 2020 (Sunday). Each day, participants in both conditions completed a short morning survey that was sent *via* WeChat App in the morning (8 am), including audio instructions for the practice, a mindfulness manipulation check, sleep quantity, sleep quality, and caffeine intake in the previous day. On day 12, participants completed a brief survey in which they reported their previous night’s sleep, caffeine intake, and Alipay account. After the completion of the study, all participants were debriefed and were invited to a daily group practice at 10 am for a 10-min mindfulness practice. This was done to ensure that all participants, including those in the mind-wandering condition, could benefit from the practice.

***State mindfulness induction***. As all our participants were native Chinese speakers, we used audio instructions in Mandarin that were recorded by a professional mindfulness coach. These instructions were developed based on well-established English mindfulness programs (Kiken and Shook, 2011). The audio instructions have been used in previous research and were effective in inducing mindfulness and mind-wandering in Chinese populations (Schuh et al., 2019). These audio instructions are available on request from the first author.

***Mindfulness manipulation check***. After listening to the audio clip in the morning, participants rated their momentary mindfulness on four items on a seven-point Likert scale (1 = not at all to 7 = Completely) (Long and Christian, 2015). Four items were “I focused on the present,” “I thought about anything I wanted (reversed coded)”, “I let my mind wander freely (reversed coded),” and “I was mindful of the present moment.”

***COVID-19 stressors***. Given that Wuhan is the city that was seriously affected by the virus before the outbreak in other cities and countries, the information about daily confirmed cases was salient to employees in Wuhan. Thus, as a proxy for the COVID-19 stressors, we recorded the number of increased cases (*M* = 384.55, *SD* = 108.28) in Wuhan between 20 February, 2020 and 1 March, 2020, from the official records of the Chinese National Health Commission of the People’s Republic of China.^1^

***Sleep quantity***. We measured sleep quantity in the survey with the following item taken from the previous studies (Lanaj et al., 2014). “How many hours of *actual sleep* did you get last night?” Recent meta-analytic research has revealed that the correlation between objective measures of sleep quantity, such as Actigraph, and self-reported measures of sleep quantity is high, indicating that self-reported measure is accurate and reliable (Litwiller et al., 2017).

***Control variables***. As a control variable, we measured participants’ trait mindfulness in the baseline survey with a 15-item scale on a 7-point Likert scale ranging from 1(never) to 7(very often) (Brown and Ryan, 2003). Fifteen items are “I could be experiencing some emotion and not be conscious of it until sometime later;” “I break or spill things because of carelessness, not paying attention, or thinking of something else;” “I find it difficult to stay focused on what’s happening in the present”, “I tend to walk quickly to get to where I’m going without paying attention to what I experience along the way;” “I tend not to notice feelings of physical tension or discomfort until they really grab my attention;” “I forget a person’s name almost as soon as I’ve been told it for the first time;” “It seems I am “running on automatic” without much awareness of what I’m doing;” “I rush through activities without being really attentive to them;” “I get so focused on the goal I want to achieve that I lose touch with what I am doing right now to get there;” “I do jobs or tasks automatically, without being aware of what I’m doing;” “I find myself listening to someone with one ear, doing something else at the same time;” “I drive places on “automatic pilot” and then wonder why I went there;” “I find myself preoccupied with the future or the past;” “I find myself doing things without paying attention”, and “I snack without being aware that I’m eating.” All items are reverse coded. (*α* = 0.79).

Consistent with sleep research, we also controlled for variables that may influence sleep quantity: sleep quality and daily caffeine intake (Gellis and Lichstein, 2009; Lanaj et al., 2014). Research has shown that poor sleep quality on one night can lead to longer sleep the next night (Banks et al., 2010). Thus, when predicting sleep quantity, we included sleep quantity and sleep quality from the previous night (i.e., lagged in time by 1 day) as control variables. We measured sleep quality with an overall item on a seven-point Likert scale ranging from 1(very bad) to 7(very good): “How do you evaluate your night’s sleep?”. We measured daily caffeine intake with one item: “Did you have beverage that contains caffeine (such as coke, coffee, etc.)?” We have also recorded daily death cases (*M* = 58.36, *SD* = 36.65) and cumulative cases (*M* = 32,658, *SD* = 3,956.85) between 20 February, 2020 and 1 March, 2020, from the official records.

### Results

Descriptive statistics and bivariate correlations are reported in Table 1. As a manipulation check, we tested whether the experimental condition had a significant effect on mindfulness (Long and Christian, 2015). Participants in the mindfulness condition reported higher levels of mindfulness than those in the mind-wandering condition (*B* = 0.29, *SE* = 0.12, *p* = 0.02), indicating that our manipulation was successful.

**Table 1:** Means, standard deviations, and correlations (Study 1).

	*Mean*	*SD*	1	2	3	4	5	6	7	8
1.Age	34.49	5.03	-							
2.Gender	1.67	0.47	0.05	-						
3.Years live in Wuhan	27.68	11.79	0.43^**^	0.05	-					
4.Experimental condition	0.57	0.50	0.06	−0.22^*^	−0.04	-				
5.Trait mindfulness	4.81	0.69	0.06	0.03	−0.20^*^	0.02	-			
6.Sleep quantity	460.60	57.66	0.05	−0.07	0.14	0.15	−0.20	-		
7.Sleep quality	4.67	0.98	0.07	0.05	0.20^*^	−0.04	0.00	0.31^**^	-	
8.Caffeine abstinence	0.73	0.36	0.10	−0.07	−0.15	0.08	0.16	0.05	0.01	-

*n* = 97 participants. Gender: 1 = male, 2 = female. Caffeine abstinence: 0 = taking caffeine, 1 = no taking caffeine. Experimental condition: 0 = mind-wandering control group, 1 = mindfulness intervention group. ^*^p < 0.05; ^**^p < 0.01.

**Figure 2:**
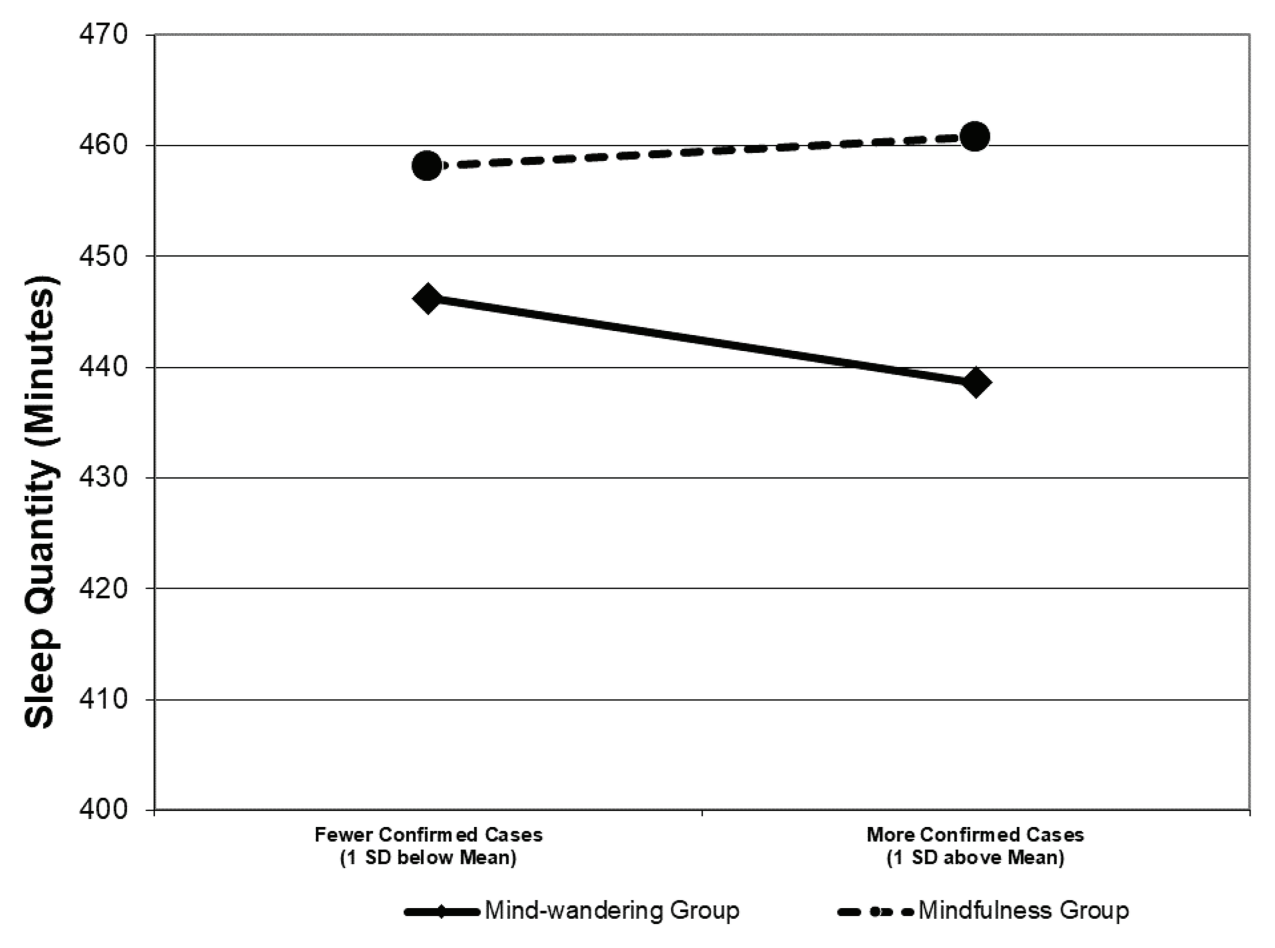
This figure visually depicts how daily mindfulness practice mitigated the relationship between COVID-19 stressors and sleep quantity in Study 1.

Given the nested nature of the data (daily observations nested within individuals), we used a multilevel modeling approach to test our hypothesis – whether the mindfulness practice would mitigate the effect of the number of daily confirmed cases on sleep quantity. Specifically, we analyzed the data with random coefficient modeling (RCMs; Raudenbush and Bryk, 2002), in which we specified the within-individual-level relationship between the number of daily confirmed cases and sleep quantity as a random slope and used the between-individual-level mindfulness intervention to predict this slope. As shown in Table 2, the mindfulness practice positively predicted the random slope between daily confirmed cases and sleep quantity (*B* = 0.05, *SE* = 0.03, *p* = 0.046). To further probe into the effect of the mindfulness practice, we plotted the simple slopes for the mindfulness treatment group and the mind-wandering treatment group, respectively. As shown in Figure 2, among people assigned to the mind-wandering group, the number of confirmed cases on a day was negatively related to their sleep quantity on that day (*B* = −0.04, *SE* = 0.01, *p* = 0.003). On average, they lost 39 min of sleep with every thousand confirmed cases reported in the city. In contrast, among people assigned to the mindfulness practice condition, their sleep quantity was unaffected by the number of confirmed cases (*B* = 0.01, *SE* = 0.01, *p* = 0.30). Hypothesis 1 was thus supported.

**Table 2:** Multilevel regression results (Study 1).

Predictors	DV = Daily Sleep quantity (Minutes)		DV = Slope between daily confirmed cases and sleep quantity	
	Estimate	*SE*	Estimate	*SE*
Intercept	324.75^**^	14.63	−0.01	0.03
***Within-individual level***				
Self-perceived sleep quality	26.41^**^	2.23		
Caffeine abstinence	8.64	6.60		
***Between-individual level***				
Experimental condition	17.00	11.67	0.05^*^	0.03

The effects of self-perceived sleep quality and caffeine abstinence on sleep quantity were modeled as fixed slopes. Caffeine abstinence was coded as 0 = taking caffeine, 1 = no taking caffeine. Experimental condition was coded as 0 = mind-wandering control group, 1 = mindfulness intervention group. ^*^p < 0.05; ^**^p < 0.01.

To check the robustness of our findings, we included sleep quantity and sleep quality on the previous night (Banks et al., 2010), trait mindfulness (Hülsheger et al., 2014), daily cumulative confirmed cases to that date, with daily cases of death reported as control variables. We found that the positive relationship between the mindfulness practice and the random slope between daily confirmed cases and sleep quantity was robust (*B* = 0.05, *SE* = 0.02, *p* = 0.04) with all these factors controlled for (see Table 3). In addition, mindfulness practice did not moderate the relationship between COVID-19 stressors and sleep quality (*B* = 0.001, *SE* = 0.001, *p* = 0.73).

**Table 3:** The robustness check results (Study 1).

Predictors	DV = Daily Sleep quantity (Minutes)		DV = Confirmed cases-sleep quantity slope	
	Estimate	*SE*	Estimate	*SE*
Intercept	225.10	414.61	0.18^*^	0.08
***Within-individual level***				
Daily cumulative cases	7.80	38.70		
Daily cases of death	0.013	0.105		
Previous day’s sleep quality	−2.79	2.78		
Previous day’s sleep quantity	0.061	0.046		
Self-perceived sleep quality	26.68^**^	2.26		
Caffeine abstinence	9.83	6.49		
***Between-individual level***				
Trait mindfulness			−0.04^*^	0.02
Experimental condition			0.05^*^	0.02

This table shows that the results of the multilevel regression with trait mindfulness, daily cumulative cases, daily cases of death, and sleep quality and quantity on the previous day as additional control variables. Caffeine abstinence was coded as 0 = taking caffeine, 1 = no taking caffeine. Experimental condition was coded as 0 = mind-wandering control group, 1 = mindfulness intervention group. ^**^p < 0.01; ^**^p < 0.05.

## Study 2

### Participants

All participants were recruited through the online platform Prolific (Palan and Schitter, 2018). Participants were pre-screened to ensure that they were (a) working full-time throughout the study and (b) working in the United Kingdom. Further, to ensure data quality, all participants had an approval rating of 95% (or above) for past studies completed on Prolific (Keith et al., 2017). Based on this, a total sample of 140 participants (59.3% female) was obtained, with a mean age of 34.1 (*SD* = 9.10), 75.1% have a college/bachelor degree, and 28.3% have a Master/PhD degree.

### Procedure

The study took place over a 2-week period with 10 surveys sent out on 10 consecutive workdays between June 8, 2020 and June 19, 2020. To be eligible for this study, participants had to complete a demographic information pre-survey. Each daily survey was emailed to participants in the evening after a typical UK workday had ended (5 pm), and this survey then expired each day at midnight. Participants were paid for each survey (GBP 1 for 5 min) along with a bonus payment for completing nine or more surveys (GBP 3). This resulted in a high completion rate with participants completing 1,302 of the 1,400 daily surveys sent out (93%).

### Measures

***COVID-19 stressors***. Unlike the early outbreak in Wuhan captured in Study 1, daily case numbers were less prominent in the UK during the data collection period since the COVID-19 pandemic has widely spread to many countries. Thus, consistent with previous studies that measure stressors (Wang et al., 2010), we operationalized COVID-19 stressors as a subjective measure that directly captures the extent to which people perceive COVID-19 as a stress that interferes with their work on a daily basis. To measure this construct, a daily measure of family-to-work conflict (see Wang et al., 2010) was adapted by supplanting the terms “home-life” *or* “family” with “COVID-19”. Items included “Today at work, how often did COVID-19 interfere with your job or career?”, “Today, how often did you think about COVID-19 related problems?”, “Today, how often did COVID-19 interfere with your responsibilities at work, such as getting to work on time, accomplishing daily tasks, or working overtime?”, “Today, how often did COVID-19 keep you from spending the amount of time you would like to spend on job or career-related activities?”, and “Today, how often did you think about things you need to do related to COVID-19?” (*α* = 0.93) In the analyses, this measure was lagged to represent the previous day’s COVID-19 stressors.

***Daily state mindfulness***. Individuals’ state mindfulness was measured on a daily level using an abbreviated version of the MAAS (Brown and Ryan, 2003) which had been previously adapted by Liang et al. (2018). Items included, “Today, I rushed through activities without being attentive to them.”, “Today, I did things without paying attention.”, “Today, I was preoccupied with thoughts of the future or the past.”, “Today, I did things automatically, without being aware of what I was doing.”, and “Today, I found it difficult to stay focused on what was happening in the present moment.” (*α* = 0.89); of note, all items are reverse-coded. In the analyses, this measure was lagged to represent the previous day’s mindfulness.

***Sleep quantity***. As in study 1, we measured sleep quantity with the following item (Lanaj et al., 2014); “How many hours of *actual sleep* did you get last night?”

***Work engagement***. Daily work engagement was measured using an abbreviated 5-item version of the Utrecht Work Engagement Scale (Schaufeli et al., 2006). Items included, “Today at work, I felt bursting with energy.”, “Today at work, I felt strong and vigorous”, “Today, I was enthusiastic about my job”, “Today, my job inspired me”, and “Today, I was immersed in my work” (*α* = 0.89).

***Control Variables***. Consistent with the past-sleep research (Gellis and Lichstein, 2009; Lanaj et al., 2014), and Study 1, we measured sleep quality as a control variable. Sleep quality was measured with an overall item on a 7-point Likert scale ranging from 1(very bad) to 7(very good): “How would you rate your sleep quality overall last night?”

### Results

Descriptive statistics and bivariate correlations for all study variables are provided in Table 4.

**Table 4:** Means, standard deviations, and correlations (Study 2).

	*Mean*	*SD*	1	2	3	4	5	6
1.Age	34.1	9.10	-					
2.Gender	1.62	0.49	−0.20^*^	-				
3.COVID-19 stressors	1.95	0.88	−0.01	0.13	-			
4.State mindfulness	4.86	0.98	0.23^**^	−0.05	−0.33^**^	-		
5.Sleep quantity	392.12	53.28	−0.21^*^	0.21^*^	−0.16	0.17^*^	-	
6.Work engagement	3.56	1.04	0.06	−0.12	−0.06	0.44^**^	0.04	-

*n* = 140 participants. Gender: 1 = male, 2 = female. ^*^p < 0.05; ^**^p < 0.01.

As in Study 1, to account for the nested nature of the data, we used multilevel modeling and centered all predictors around each participant’s mean score (Hofmann et al., 2000). However, because we focused on daily state mindfulness (vs. an individual-level mindfulness practice) in this study and tested only within-individual (vs. between-individual) effects, we used fixed slope modeling. The relationships of COVID-19 stressors, state mindfulness, and their interaction with sleep quantity were all modeled as fixed slopes. Given that variables such as trait mindfulness, sleep quality, and daily caffeine intake did not influence results in any way in Study 1, we did not control for these variables in our analyses.

**Figure 3:**
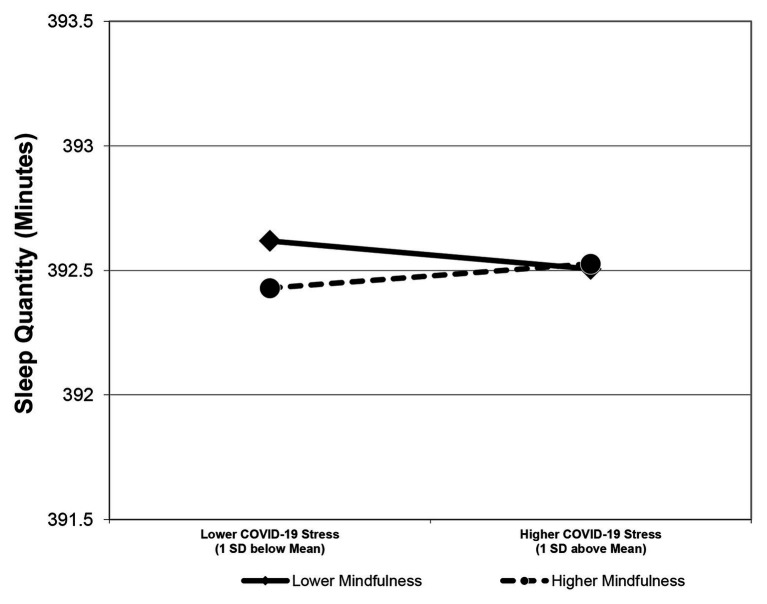
This figure visually depicts how daily state mindfulness mitigated the relationship between COVID-19 stressors and sleep quantity in Study 2.

Consistent with Hypothesis 1, the interactive effect of daily COVID-19 stressors and mindfulness on sleep quantity was positive and significant (*B* = 0.11, *SE* = 0.05, *p* = 0.04, see Table 5). Simple slope analyses further revealed that the relationship between COVID-19 stressors and sleep quantity was negative when state mindfulness was low (*B* = −0.11, *SE* = 0.05, *p* = 0.03) and nonsignificant when state mindfulness was high (*B* = 0.09, *SE* = 0.05, *p* = 0.20); difference in simple slopes = 0.20, *SE* = 0.10, *p* = 0.04; Figure 3). Mindfulness neutralized the negative effect of COVID-19 stressors on sleep duration; Hypothesis 1 was thus supported. In addition, results indicated that mindfulness did not moderate the relationship between COVID-19 stressors and sleep quality (*B* = 0.10, *SE* = 0.06, *p* = 0.07).

**Table 5:** Multilevel regression results in (Study 2).

Predictors	DV = Sleep quantity	
	Estimate	*SE*
COVID-19 stressors	−0.007	0.084
State mindfulness	−0.046	0.037
COVID-19 stressors × State mindfulness	0.109^*^	0.052

^*^p < 0.05; ^**^p < 0.01.

We continued to test a moderated mediation model in which state mindfulness moderated an indirect effect of COVID-19 stressors on work engagement *via* sleep quantity (H2). Analyses revealed that there was a positive relationship between sleep quantity and work engagement (*B* = 0.14, *SE* = 0.03, *p* < 0.01). With a Monte Carlo simulation method (Preacher et al., 2010), we found that the indirect effect of COVID-19 stressors on work engagement *via* sleep quantity was negative and significant when state mindfulness was low [estimate = −0.01, 95% *CI* = (−0.030, −0.002)] and nonsignificant when state mindfulness was high [estimate = 0.01, 95% *CI* = (−0.001, 0.027); difference in conditional indirect effects = 0.03, 95% *CI* = (0.002, 0.059)]. Hypothesis 2 was thus supported.

## General Discussion

We found that induced or measured state mindfulness significantly buffered the negative effect of COVID-19 stressors on sleep duration (Studies 1 and 2) and work engagement (Study 2) such that COVID-19 stressors had negative effects when state mindfulness was low while negative effects were buffered when state mindfulness was high. Findings of the present studies contribute to the employee stress and well-being research as well as the emerging mindfulness research in the organizational literature.

First, our work extends the recovery literature by looking beyond the widely examined organizational factors and introducing a non-organization factor that is probably the most powerful external force that disrupts employees’ sleep. Previous studies have shown that organizational factors such as occupational stressors and work-family conflict can harm employees’ sleep (DeArmond and Chen, 2004; Blau, 2011; Barnes et al., 2012; Lanaj et al., 2014; Berkman et al., 2015). Understanding these organizational antecedents can help employees and organizations achieve better recovery by specifically alleviating these potential stressors. Unlike organizational factors, traumatic events such as the COVID-19 pandemic are external shocks that are not controllable by organizations and employees. Yet, it has detrimental effects on employees’ sleep (Sinha, 2016). Indeed, a recent review suggests that “scholars should consider how recovery, an inherently non-work activity, is impacted by non-work demands and resources in addition to work-specific demands and resources” (Steed et al., 2019; p.24). Our research echoes this view and highlights the importance of considering public traumatic events when examining the employees’ recovery process.

Second, organizational research on sleep has generally used one of the two indicators to capture sleep: sleep quality and sleep quantity (Barnes, 2012; Litwiller et al., 2017). Although they are conceptually similar and related, they are different because they tend to be correlated with different variables (Barclay et al., 2010; Hülsheger et al., 2015; Litwiller et al., 2017). Consistent with previous studies, our findings that mindfulness interacts with COVID-19 stressors to influence sleep quantity, but not quality, further confirms that they are two distinct concepts (Litwiller et al., 2017). Specifically, compared to sleep quality, sleep quantity is more closely related to resources available for work, which have downstream effects on work engagement (Lanaj et al., 2014). Indeed, we show that sleep quantity is a crucial mechanism through which mindfulness exerts a buffering effect on work engagement. Thus, our findings have important implications for different roles that sleep quality and quantity may play in work-related outcomes.

Third, our work extends the employee stress and well-being research by looking beyond the widely examined intra-organizational stressors and examining the negative effects of acute-extraorganizational stressors. Research on employee stress and well-being dates back nearly 100 years, during which traumatic events such as World War I, the influenza epidemic of 1918, and the Great Depression have greatly impacted employees (see Bliese et al., 2017 for a recent review). Although the origins of the field were stimulated by these events, the majority of research to date has focused on the effect of intra-organizational stressors. However, growing evidence has mounted, showing that acute-extraorganizational stressors play an equally significant role in impacting employees’ outcomes, e.g., increasing absenteeism and decreasing job satisfaction, and leading to higher turnover (Byron and Peterson, 2002; Hochwarter et al., 2008; Dollard et al., 2013; Ragins et al., 2014; Baruch et al., 2016). Nevertheless, research on how to tackle these negative effects has lagged behind (Byron and Peterson, 2002; Hochwarter et al., 2008). Furthermore, the few interventions that have been introduced in the psychology literature were conducted after the crisis occurred and focused on “fixing” *post-traumatic effects* rather than timely intervening negative effects of the crisis as it unfolds (Lavie, 2001; Sinha, 2016). This post-hoc approach is at odds with recent research, suggesting that the early treatment of trauma-induced stress may be more effective in preventing the development of post-traumatic negative experiences such as depression (Sinha, 2016). Therefore, our study is among the first to examine how the negative impacts of extra-organizational stressors can be neutralized *during a crisis*. In doing so, we identified that mindfulness, both as a state and implemented as a randomized-controlled intervention, is effective in mitigating the negative effect of an ongoing crisis on an employee’s well-being. Thus, our work has general theoretical implications for managing acute-extraorganizational stressors.

Furthermore, our study contributes to organizational research on mindfulness. Past work has found mindfulness can be an effective intervention in workplace settings providing a myriad of positive effects on work attitudes and outcomes (for reviews see Sutcliffe et al., 2016; Kay et al., 2019). Moving beyond these main effects of mindfulness, this paper identifies mindfulness as an effective crisis intervention. Specially, this paper draws on the metacognitive theory of mindfulness and extends this theory into the context of crisis by showing that mindfulness can neutralize the negative effects of COVID-19 stressors on work engagement *via* the mediating role of sleep duration, a mechanism that is highly vulnerable to the hyperarousal state triggered by trauma. Further, this paper also contributes to growing research on the role of state mindfulness in the workplace (e.g., Tuckey et al., 2018; Hafenbrack et al., 2020), investigating this through a low-dose intervention along with measuring it as a self-reported state. The synergy of these results provides promise for future research interested in examining the daily impacts of mindfulness along with providing a low-cost (or even free) intervention for organizations to implement in the light of acute extra-organizational stressors.

Our research also has practical implications. In uncertain times like the COVID-19 pandemic, how organizations treat their employees will have a lasting impact on employees’ loyalty, engagement, and productivity (Carvalho and Areal, 2016). Our findings suggest that mindfulness practice can be introduced as an effective employee care program for organizations. Importantly, our findings provide further evidence that even a “low dose” of on-line mindfulness practice is effective (Hülsheger et al., 2015). Thus, during the COVID-19 outbreak, organizations that offered morning meditations to all company employees (e.g., Google), might have been more effective in managing the negative impacts of the pandemic on employee engagement.

### Limitations and Future Research Directions

Despite several contributions to the literature, the present study should be viewed in light of its strengths and weaknesses. First, we argue that mindfulness can activate a metacognitive process of observing thoughts without judgment, thus reducing the secondary arousal related to trauma. While this research argumentation is consistent with the neuroscience literature on mindfulness and metacognition (Jankowski and Holas, 2014), we did not examine the specific psychological mechanisms that underlie this effect. To date, studies have examined mechanisms such as cognitive reappraisal, decentering process, and affective rumination for the effects of mindfulness (Fresco et al., 2007; Liang et al., 2018; Kay and Skarlicki, 2020). In addition, it is also possible that COVID-19 stressors are associated with increased workload, alternative shifts, interpersonal conflict. These are possible mechanisms of mindfulness on improving sleep duration. Thus, we suggest that future research could examine the exact mechanisms in the moderating effects of mindfulness on sleep duration.

Second, our research examines the neutralizing of mindfulness in the relationship between the COVID-19 stressors and employees’ sleep duration and work engagement. However, post-traumatic growth theory suggests that it is possible that individuals can benefit and grow from traumatic exposure (Tedeschi and Blevins, 2015). Specifically, this theory suggests that mindfulness may facilitate positive reappraisal of the negative experience. In line with the mindfulness-to-meaning hypothesis (Garland et al., 2017), this reappraisal process can in turn make individuals experience growth in aspects such as personal strength and appreciation of life. Future research should take a growth perspective and examine the effect of mindfulness on individuals’ post-traumatic growth.

Third, a strength of this study was the dual operationalization of mindfulness through a randomized-control trial and a daily self-reported measure. Organizational studies typically focus on a sole operationalization, despite scholars often referring to mindfulness state, trait, and trained skill being a unitary construct (Reb et al., 2020). Therefore, the replication of our results using two operationalization provides further evidence for the generalized effect of mindfulness. Nevertheless, the moderation graphs for Study 1 and 2 have slight discrepancies, suggesting that there might be fine grained difference between the operationalization. However, despite the discrepancy, it is noteworthy that both studies support our primary hypothesis that mindfulness will neutralize the negative effect of COVID-19 stressors, demonstrated by simple slope analyses, showing that COVID-19 *only* has a significant negative effect on sleep quantity when mindfulness is low. This helps contribute to the literature linking mindfulness with sleep outcomes (Ong et al., 2012; Ong and Smith, 2017); nevertheless, there are still important questions for the field to address. One key question that arises as a result of the current study is understanding how sleep quantity (and quality) impacts mindfulness. This study, and the majority of past work (for a review see Ong and Smith, 2017), has focused on how mindfulness impacts sleep but given that practicing mindfulness has its own self-regulatory challenges (Mrazek et al., 2020), it is possible that a good night’s sleep could increase an individual’s capacity to engage in, and thus benefit from, mindfulness practice. Building on the past work, this would suggest a potentially virtuous cycle in which mindfulness improves sleep and then better sleep subsequently improves mindfulness.

Furthermore, consistent with previous studies (Barnes, 2012), our research confirms that sleep quality and sleep quantity are two distinct concepts. Research suggests that sleep quantity is more closely related to resources available for work while sleep quality is more closely related to employees’ perceptions or emotions (Litwiller et al., 2017). Indeed, we show that sleep quantity is a crucial mechanism through which mindfulness exerts a buffering effect on work engagement. This is because sleep quantity provides resources for employees to be engaged in the workplace. Future research should take into consideration other work-related outcomes that are related to perceptions, such as job satisfaction and negative affect, and examine the buffering effect of mindfulness on these perceptual outcomes.

## Data Availability Statement

The raw data supporting the conclusions of this article will be made available by the authors, without undue reservation.

## Ethics Statement

The studies involving human participants were reviewed and approved by Study 1 was approved by the Ethics committee at China Europe International Business School (CEIBS). Study 2 was approved by the Ethics committee at the National University of Singapore. The participants provided their written informed consent to participate in this study.

## Author Contributions

MZ and JN designed Study 1. MZ collected the data for Study 1. JN, TM-W, YL, and NT designed Study 2. JN, TM-W, and YL collected the data for Study 2. JY, MZ, and TM-W analyzed the data from both studies. MZ, TM-W, and JN wrote first drafts of the paper. All authors provide comments and inputs. All authors contributed to the article and approved the submitted version.

### Conflict of Interest

The authors declare that the research was conducted in the absence of any commercial or financial relationships that could be construed as a potential conflict of interest.

## Footnotes

^1^http://www.nhc.gov.cn/wjw/xinx/xinxi.shtml
